# Onset of psychosis at age 81? With regard to frontal lobe syndromes

**DOI:** 10.1590/S1679-45082015RC3004

**Published:** 2015

**Authors:** Patrícia Pedro, Diogo Telles-Correia, Iolanda Godinho, Carlos Chagas

**Affiliations:** 1 Centro Hospitalar Psiquiátrico de Lisboa, Lisboa, Portugal.; 2 Hospital de Santa Maria, Lisboa, Portugal.; 3 Hospital Pulido Valente, Lisboa, Portugal.

**Keywords:** Frontal lobe/pathology, Aged, Conduct disorder, Psychopathology, Case reports

## Abstract

When the frontal lobe of the brain is affected important behavioral changes may occur mainly at the level of executive functioning, *i.e.*, planning, decision-making, judgment and self-perception. However, the behavioral changes may be of different nature with marked indifference and apathy. We report a clinical case of an 81-year-old patient with sudden onset of behavioral changes that were initially interpreted as an acute confusional episode of infectious etiology, but actually they were due to an ischemic lesion in the frontal lobe.

## INTRODUCTION

The frontal lobe is the largest lobe and it is linked, in a complex manner, to multiple regions of the brain. Injury to frontal bone can lead to variety of clinical presentations. However, this injury assessment is often neglected in routine neurological exams and also in tests for mental state examination to test frontal lobe function such as Mini Mental State Examination (MMSE). The Frontal Assessment Battery (FAB) and Montreal Cognitive Assessment (MoCA^©^) are other tests that can be useful to evaluate frontal lobe function.^(1,2)^

When frontal lobe is affected changes may occur in the behavior particularly in executive functions, such as planning, decision making, judgment and self-perception.^(2,3)^ The most common form is characterized by emotional liability and changes in social judgment and impulse control.

Other changes are characterized by marked indifference and apathy. The individual can lose the interest for his/her daily activities and seem indifferent to surrounding facts. Both cases can be associated with frontal lobe injuries (the first, injury to orbitomedial and the second, injury to the dorsolateral prefrontal) named as frontal lobe syndrome.^(4)^

Patients with frontal dorsolateral cortex injuries can also have little working memory for verbal information (if the left hemisphere is significantly affect) or for spatial information (if right hemisphere is the most injured area).^(2)^

Injuries in the inferotemporal circuit can be associated to deficits in visual discrimination, visual hallucination and psychosis. Frontal lobe syndromes can present several etiologies: mental delay, traumatic brain injuries, brain tumors, degenerative dementia, cerebrovascular disease, normal pressure hydrocephalus, schizophrenia, depression, among others.^(1,2)^

The major differential diagnoses to frontal lobe syndrome are urinary tract infection, septicemia, hipo or hyperglycemia, thiamine deficiency, among other. In addition, it may be associated with abuse or abstinence of drugs and iatrogenesis, particularly sedative or anticholinergic drugs. Neurological conditions should be equally considered, such as, cranioencephalic traumatism, epilepsy or encephalitis. Other general causes, *i.e.*, obstipation, dehydration, pain or sensorial privation should be also investigate.^(5)^

## CASE REPORT

We report a case of an 81-year-old widow (MNF), autonomous for daily life activities and with history of hypertension and dyslipidemia. She was referred to family physician because of sudden change on her behavior such as isolation, psychomotor agitation, hetero-aggressiveness, anomie, visual/auditory verbal hallucinations, persecutory deliriums and total insomnia. The computed tomography scan of the brain showed large periventricular parietal focal atrophy in the left side. Lacunar stroke internal capsule and left thalamic lacunar stroke. It also showed diffuse cortical and subcortical cerebral atrophy with predominance in frontal and temporal internal to left side, mural atheromatosis calcification of the carotid siphons, and inflammation of left middle ear. She was referred to emergency service and her exams showed a mild neutrophilia of 8,950x10^9^/L, CRP test of 2.8mg/dL, urine II with leukocyturia (70cell/uL), erythrocyturia (25cell/uL) and negative nitrite test. The computed tomography scan of the brain was redone and no *de novo* alterations were seen. The patient was discharged and risperidone 1mg daily was prescribed. In the night after discharge she had total insomnia.

She returned to the emergency service and remained hospitalized for one hour for surveillance. She was debilitated, extremely sleepy and had little reaction to stimulus, including painful stimulus. The CRP test was repeated and it was 1.8mg/dL. In the subsequent day, the patient was vigil and had psychomotor agitation. In addition, she was disoriented in time and space in time and space, self and allopsychically oriented, disinhibited, sometimes presented caught, but not fixed, attention, had fluent and spontaneous discourse, but incoherent responses. The patient also had loose association and audio and visual hallucination.

The suspicion diagnosis was acute confusional state and the neurologist’s opinion was requested. The neurologist did not observe significant alterations in neurological exam, no deficits in cranial partners, strength, sensibility or limbs coordination, and also no meningeal signs.

For exclusion of meningitis/encephalitis with eventual onset point in middle hearing, a lumbar puncture was performed and revealed a limpid aspect liquor with normal cytochemical characteristics. She returned to psychiatry and repeated the analyses that revealed leukocytosis 17,500x10^9^/L with neutrophil (85.68%), CRP test 4.66mg/dL, urine II with leukocyturia (17cell/uL), erythrocyturia (37cell/uL) and negative nitrite test and normal thyroid functions.

The internal medicine department interpreted laboratorial findings as possible non-complicated cystitis. The patient was hospitalized for etiologic investigation and treatment. A significant increase of CRP was seen for 12.3mg/dL and, because of slight changes in urine II, an antibiotic therapy was introduced with cefhiaxone after the urine culture collection that was negative. She underwent the Venereal Disease Research Laboratory (VDRL) test, which was negative, and underwent replacement of thiamine so that admitting deficit of vitamin.

In order to exclude other non-urinary infectious causes due to the increase of inflammatory parameters, the patient underwent blood culture with negative results and echocardiogram and thoracic-abdominal-pelvic CT scan, which revealed no significant changes (Chart 1).

**Chart 1:** Complementary exams and diagnosis

An 81-year-old women with sudden change on behavior, isolation, psychomotor agitation, hetero-aggressiveness, anomie, visual/auditory verbal hallucinations, persecutory deliriums and total insomnia					
Diferential diagnosis			Organic clinical picture versus psychiatric clinical picture		
Computed tomography scan of the brain			Inflammation of left middle ear		
Neurology			No focal sings in neurologic exam		
Lumbar puncture			Limpid aspect liquor and with normal cytochemical characteristics		
Analyses			Leukocytosis (17.500x10^9^/L) with mild neutrophilia (85,68%), high CRP test of (4.66mg/dL → 12.3mg/dL)		
			Urine II with leukocyturia (17 cell/uL) and erythrocyturia (37 cell/uL)		
Hospitalized patient with suspicion of uncomplicated cystitis					
Antibiotic therapy with cefriaxone			Negative urine culture		
Exclusion of no urinary tract causes for inflammatory/confusion clinical feature					
Negative VDRL	Negative blood culture	Ecocardiograpm without changes		Normal thyroid function	Thoracic-abdominal-pelvic CT scan without signficant changes
Maintenance of behavioral changes					
Cranioencefálico magnetic resonance imaging			Cortical and subcortical frontal internal injury compatible with possible subacute failure		

VDRL: Venereal Disease Research Laboratory; TAP- CT: thoracic-abdominal-pelvic computed tomography scan.

For maintenance of behavioral changes, a cranioencephalic magnetic resonance imaging was performed and revealed a “parafacial internal frontal cortical-subcortical injury in convexity to the right, measuring about 21mm in diameter, which is compatible with possible subacute failure”. We begin administration of captopril 12.5mg 3id, acetylsalicylic acid (AAS) 250mg 1id and rosuvastatin 10mg 1id, in addition psychiatric drugs were adjusted (quetiapine 25 1id).

The patient had progressively improvement. At discharge she was oriented in time, but not in space, had an euthymic mood, no visual/auditory verbal hallucinations, no changes on format and content of thinking, caught and fixed attention, coherence and spontaneous discourse, insight and critical judgment. She was discharged and referred to her family physician and to a psychiatrist.

## DISCUSSION

This study main clinical findings (sudden behavioral changes in an old woman) and laboratorial tests (Leukocytosis with mild neutrophilia, high CRP, urine II with leukocyturia and erythrocyturia) lead the hypothesis of urinary infection. The patient was admitted to internal medicine service with hypothesis of uncomplicated cystitis. Her urine culture was negative and no improvement was seen in clinical picture after administration of antibiotic therapy. In order to exclude other non-urinary infections due to the increase in inflammatory parameters, more complementary exams were requested for the diagnosis. Of these the only one that presented changes was the cranioencephalic magnetic resonance imaging that revealed “fronta cortical-subcortical injury […] compatible with possible subacute failure”.

Sudden change of mental status, disorientation, fluctuations of consciousness, difficult to get focused and keep attention, sleep-alertness disorders, psychomotor agitation and hallucination (typically visual) led to the exclusion of organic cause, namely cerebrovascular. Other clinical pictures exist and they that can lead to the same type of symptoms, but in more insidious form: dementia, psychotic affective disorders.^(6)^

In this case, the uncommon localization of the ischemic event not detected in cranioencephalic magnetic resonance imaging, the lack of focal deficits and changes in other inflammatory parameters can lead to no diagnostic suspicion of frontal lobe syndrome.

## CONCLUSION

This case reinforces the importance to suspect and investigate the etiology of acute confusional states, especially in older population, when the diagnosis is challenging.
