# Supplementary material for: Onset of psychosis at age 81? With regard to frontal lobe syndromes
Source: Einstein (Sao Paulo). 2015 Oct-Dec;13(4):615–7. doi: 10.1590/S1679-45082015RC3004 (PMC4878641; doi:10.1590/S1679-45082015RC3004)
Supplement: Translation

# Translation: Psicose inaugural aos 81 anos? A propósito das síndromes do lobo frontal

**Authors:** Patrícia Pedro, Diogo Telles-Correia, Iolanda Godinho, Carlos Chagas

**Affiliations:** 1 Centro Hospitalar Psiquiátrico de Lisboa, Lisboa, Portugal.; 2 Hospital de Santa Maria, Lisboa, Portugal.; 3 Hospital Pulido Valente, Lisboa, Portugal.

**Keywords:** Lobo frontal/patologia, Idoso, Transtorno da conduta, Psicopatologia, Relatos de casos

## Abstract

O lobo frontal, quando afetado, pode provocar alterações importantes do comportamento, principalmente na função executiva: planejamento, tomada de decisões, juízo e autopercepção. Contudo, as alterações podem ser de outra natureza, caracterizando-se por marcada indiferença e apatia. É relatado aqui o caso de uma paciente de 81 anos, com alterações súbitas do comportamento, que foram interpretadas inicialmente como um quadro confusional agudo de natureza infecciosa, mas que se mostraram provocadas por uma lesão isquêmica na região frontal.

## INTRODUÇÃO

O lobo frontal é o maior e liga-se de forma complexa a múltiplas regiões do cérebro. Quando afetado, pode levar a uma multiplicidade de apresentações clínicas. Contudo, com frequência não é avaliado especificamente nos exames neurológicos de rotina e nem testes utilizados para avaliar o estado mental, como o *Mini Mental State Examination* (MMSE), não foram concebidos para testar a função do lobo frontal. O *Frontal Assessment Battery* (FAB) e o *Montreal Cognitive Assessment* (MoCA^©^) são outros testes que podem ser úteis para avaliar do lobo frontal.^(1,2)^

Quando o lobo frontal é afetado, podem ocorrer alterações do comportamento, nomeadamente das funções executivas: planejamento, tomada de decisões, juízo e autopercepção.^(2,3)^ A forma mais comum caracteriza-se por labilidade emocional e alterações no controle do impulso e do juízo social.

Também pode ocorrer outra forma, caracterizada por marcada indiferença e apatia. O indivíduo pode perder o interesse por suas atividades habituais e parecer indiferente aos acontecimentos ao seu redor. Ambos os casos podem estar associados a lesão dos lobos frontais (o primeiro, com lesões orbitomediais e o segundo, com lesões pré-frontais dorsolaterais), sendo referidos como síndromes do lobo frontal.^(4)^

Os doentes com lesões no córtex frontal dorsolateral podem também apresentar pouca memória de trabalho para informação verbal (se o hemisfério esquerdo está predominantemente afetado) ou para a informação espacial (se o hemisfério direito é o mais lesionado).^(2)^

Lesões a nível do circuito inferotemporal podem estar associadas a défices na discriminação visual, alucinações visuais e psicose. As síndromes do lobo frontal podem ter várias etiologias: atraso mental, lesão cerebral traumática, tumores cerebrais, demências degenerativas, doença cerebrovascular, hidrocefalia de pressão normal, esquizofrenia, depressão, entre outras.^(1,2)^

Os principais diagnósticos diferenciais relativamente à síndrome do lobo frontal são condições médicas como, por exemplo, infecção do trato urinário, septicemia, hipo ou hiperglicemia, deficiência de tiamina, entre outras, além de abuso ou abstinência de drogas e iatrogenia, particularmente com medicamentos sedativos ou anticolinérgicos. Devem ser consideradas igualmente condições neurológicas, como, por exemplo, traumatismo craniencefálico, epilepsia ou encefalite. Outras causas mais gerais, como obstipação, desidratação, dor ou privação sensorial, também devem ser investigadas.^(5)^

## RELATO DE CASO

Paciente (MNF) com 81 anos de idade, viúva e autônoma para as atividades da vida diária, com antecedentes de hipertensão arterial e dislipidemia. Foi levada ao seu médico de família por alteração súbita do seu comportamento, apresentando isolamento, agitação psicomotora, heteroagressividade, anomia, alucinações visuais e auditivo-verbais, delírios persecutórios e insônia total. Fez tomografia computadorizada craniencefálica que revela “áreas de atrofia focais periventriculares parietais de maiores dimensões à esquerda. Enfartes lacunares núcleo-capsulares e talâmico esquerdo. Padrão de atrofia córtico-subcortical encefálica difusa de predomínio frontal e temporal interno à esquerda. Calcificações ateromatosas murais nos sifões carotídeos. Preenchimento inflamatório do ouvido médio esquerdo.” Recorreu ao serviço de urgência, onde faz análises que revelam ligeira neutrofilia de 8.950x10^9^/L, PCR de 2,8mg/dL, urina II com leucocitúria (70cel/uL), eritrocitúria (25cel/uL) e pesquisa de nitritos negativa. Repete tomografia computadorizada craniencefálica que não revelou alterações de novo. Tem alta medicada com risperidona 1mg uma vez por dia. Manteve-se agitada e com insônia total naquela noite.

Regressa ao serviço de urgência, onde passou uma noite no serviço de observação, prostrada, muito sonolenta e pouco reativa a estímulos, incluindo dolorosos. Repetiu análises que mostram PCR de 1,81mg/dL. No dia seguinte, a paciente encontrava-se vigil, com agitação psicomotora, desorientada no tempo, espaço, auto e alopsiquicamente, desinibida, com atenção por vezes captável, mas não fixável, com discurso espontâneo e fluente, mas incoerente, com respostas ao lado e marcado afrouxamento das associações e atividade alucinatória auditiva e visual.

Ponderou-se a hipótese de quadro confusional agudo e pediu-se a colaboração da Neurologia, que não encontrou alterações significativas no exame neurológico, nomeadamente sem défices nos pares cranianos, na força, sensibilidade ou coordenação nos membros, e sem sinais meníngeos.

Para exclusão de meningite/encefalite com eventual ponto de partida no ouvido médio, fez-se punção lombar, com saída de liquor límpido e com citoquímica normal. Regressou à Psiquiatria e repetiu análises, que revelam leucocitose de 17.500x10^9^/L com neutrofilia (85,68%), PCR de 4,66mg/dL, urina II com leucocitúria (17cel/uL), eritrocitúria (37cel/uL), além de pesquisa de nitritos negativa e provas de função tiroideia normais.

Foi observada pela Medicina Interna, que interpretou achados laboratoriais como possível cistite não complicada, sendo internada para investigação etiológica e tratamento. Verificou-se um aumento substancial da PCR para 12,3mg/dL e, por ligeiras alterações na urina II, iniciou antibioterapia com ceftriaxona após a coleta de urocultura, que veio negativa. Efetuou o teste *Venereal Disease Research Laboratory* (VDRL), que foi negativo, e foi realizada reposição vitamínica de tiamina, admitindo-se défice vitamínico.

Na tentativa de exclusão de outras causas infecciosas não urinárias para o aumento dos parâmetros inflamatórios, a paciente realizou hemoculturas, que se verificaram negativas, e ecocardiograma e TC tóraco-abdomino-pélvica, sem alterações relevantes a salientar (Quadro 1).

**Quadro 1:** Exames complementares e marcha diagnóstica

Paciente feminina, 81 anos, alteração súbita do comportamento, isolamento, agitação psicomotora, heteroagressividade, anomia, alucinações visuais e auditivo-verbais, delírios persecutórios e insônia total					
Diagnóstico diferencial			Quadro orgânico *versus* quadro psiquiátrico		
Tomografia computadorizada craniencefálica			Sem alterações agudas. Preenchimento inflamatório do ouvido médio esquerdo		
Neurologia			Sem sinais focais ao exame neurológico		
Punção lombar			Líquor límpido e com citoquímica normal		
Análises			Leucocitose (17.500x10^9^/L) com neutrofilia (85,68%) e subida de PCR (4,66mg/dL → 12,3mg/dL)		
			Urina II com leucocitúria (17cel/uL) e eritrocitúria (37cel/uL)		
Doente internada com suspeita de cistite não complicada					
Antibioterapia com ceftriaxona			Urocultura negativa		
Exclusão de causas não urinárias para o quadro inflamatório/ confusional					
VDRL negativo	Hemoculturas negativas	Ecocardiograma sem alterações		Função tiroideia normal	TC-TAP sem alterações relevantes
Manutenção das alterações do comportamento					
Ressonância magnética craniencefálica			Lesão cortico-subcortical frontal interna compatível com possível enfarte subagudo		

VDRL: *Venereal Disease Research Laboratory*; TC-TAP: tomografia computadorizada tóraco-abdomino-pélvica.

Por manutenção das alterações do comportamento, realizou ressonância magnética craniencefálica que revelou “lesão córtico-subcortical frontal interna parafacial na convexidade à direita, medindo cerca de 21mm de maior diâmetro, compatível com possível enfarte subagudo”. Iniciou medicação com captopril 12,5mg 3id, ácido acetilsalicílico (AAS) 250mg 1id e rosuvastatina 10mg 1id, sendo feito ajuste da medicação psiquiátrica (quetiapina 25 1id).

A paciente melhorou progressivamente, apresentando-se, à data da alta, orientada na pessoa e no tempo, não orientada no espaço, com humor eutímico, sem alucinações auditivo-verbais e visuais, sem alterações da forma e do conteúdo do pensamento, com atenção captável e fixável, discurso coerente e espontâneo, com *insight *e juízo crítico. Teve alta referenciada ao seu médico de família e consulta de Psiquiatria.

## DISCUSSÃO

Os principais achados clínicos (alteração súbita de comportamento em idosa) e laboratoriais (leucocitose com neutrofilia, PCR aumentada, urina II com leucocitúria e eritrocitúria) apontavam para a hipótese de infecção urinária, sendo então a doente internada no Serviço de Medicina Interna, com a hipótese diagnóstica de cistite não complicada. Fez urocultura, que foi negativa e não se verificou melhoria do quadro clínico com antibioterapia. Na tentativa de exclusão de outras causas infecciosas não urinárias para o aumento dos parâmetros inflamatórios, fizeram-se mais exames complementares de diagnóstico, dos quais o único que apresentou alterações foi a ressonância magnética craniencefálica que revelou “lesão córtico-subcortical frontal […] compatível com possível enfarte subagudo”.

A alteração súbita do estado mental, com desorientação, flutuações do estado de consciência, dificuldade em focar e manter a atenção, distúrbio do ciclo sono-vigília, agitação psicomotora e alucinações (tipicamente visuais) obrigou a exclusão de causa orgânica, nomeadamente cerebrovascular. Existem outros quadros clínicos que podem levar ao mesmo tipo de sintomatologia, mas de forma mais insidiosa: demência, perturbações afetivas e psicóticas.^(6)^

Nesse caso, a localização pouco habitual do evento isquêmico não detectada na tomografia computadorizada craniencefálica a ausência de défices focais e as alterações de outros parâmetros inflamatórios podem ter levado à não suspeição de uma síndrome do lobo frontal.

## CONCLUSÃO

Este caso reforça a importância de suspeitar e pesquisar a etiologia de estados confusionais agudos, sobretudo em idades mais avançadas, quando o diagnóstico é particularmente desafiante.
